# Hospital and Institutional News

**Published:** 1912-10-19

**Authors:** 


### HOSPITAL AND INSTITUTIONAL NEWS.

### HOSPITALS AND NURSES IN THE WAR ZONE.

Elsewhere we deal briefly with the army ambulance service in connection with the war in the Near East. It is obvious, however, that the resources of field and emergency hospitals will be inadequate unless assisted by those of permanent institutions. As is well known, the Roumanian hospitals at Bucharest, although old and in many respects modelled on the obsolete plans of Viennese institutions, are admirably staffed and competent to cope with major war surgery. At Belgrade there is a fine military hospital and several smaller civil hospitals which will be available for use, while Greece possesses some new hospitals which should serve her in excellent stead in case she decides upon war. Turkey is comparatively well off in the matter of permanent military hospitals. At Constantinople there are also two fine modern institutions, of which one presents some interesting structural features. Bulgaria and Montenegro are much less adequately provided for in the matter of permanent hospital arrangements. Their institutions present most of the worst defects to be found in the old septic wards of the antiquated hospitals of Vienna, and much care and considerable structural alteration will be needed to make these institutions fit to cope with a large influx of acute medical or surgical cases. The greatest desideratum will undoubtedly be proper nurses. In most of the States the nursing is in charge of sisterhoods whose membership is limited, however strenuous may be the personal activity of the members themselves. Doubtless steps have been taken to meet this deficiency, but already we are informed by the war correspondents that the base hospitals are choked and that the nursing arrangements leave much to be desired. The erection of large emergency hospitals on the pavilion plan appears to be the best way out of the difficulty so far as accommodation is concerned, but the provision of suitable medical men and nurses will not be so easy. Those interested in hospitalisation in time of war will follow with considerable interest the developments in these directions in the Balkan States.

### KING'S COLLEGE HOSPITAL OLD SITE.

Many of our readers will doubtless have noted with interest the announcement in our advertisement columns last week that Messrs. Weatherall and Green are prepared to receive tenders (up to December 18) for the disposal of the site upon which King's College Hospital now stands. Vacant possession of the site and buildings will be given on January 1, 1914. The total area of ground, which is all freehold, is given as 48,154 square feet, and it constitutes practically an island site, having frontages on four streets, though none of them can be said to be main thoroughfares. With so many vacant plots of ground still to let in and about Kingsway and Aldwych, close to the King's site, the immediate disposal on advantageous terms of this ground may not be simple; but the terms (apart from actual consideration money) upon which possession will be given are probably much less onerous than those which the County Council imposes upon its tenants, and this may suffice to attract substantial tenders. The Governors of King's College Hospital announce three alternatives for tenderers. First, they will sell the freehold out right—provided, we may suppose, that they can get their price for it. Secondly, they will grant a ninety-nine years' lease, provided the lessee undertakes to expend £100,000 on new buildings to be erected on the site. Thirdly, they will grant a sixty years' lease, the lessee in that case covenanting to expend £25,000 on alterations to the existing buildings. For the sake of the hospital poor of South London we may hope that this splendid site will attract keen bidders.

### THE NOBEL PRIZE: A CRITICISM.

The announcement has been made that the Nobel Trustees have awarded the Nobel Prize for Medicine for 1912 to Dr. Alexis Currel, of the Rockefeller Institute in New York. The value of the prize this year amounts roughly to £7,800. The award states that the prize is awarded for Dr. Currel's work on the suture of vessels and the transplantation of organs. In this country surgeons have taken little part in the pioneer work of blood-vessel suture, and no one will deny that the United States bears off the honours in this branch of research. In Britain, however, the name of Matas is better known than that of the Nobel prize-winner, and the operation of endo-aneurysmorrhaphy is generally known by Matas' name. Doubtless the Nobel Trustees have been guided by the best American expert advisers in making their award, but it is certainly a drawback to the system that an enormous reward falls to one worker in a field where his colleagues of almost equal merit get nothing.

### A REVOLUTION IN RATING AT NORWICH.

Great importance was given to the quarterly meeting of the Norfolk and Norwich Hospital, which was held last Saturday, by the discussion which took place on a recent proposal of the Guardians to rate the hospital in full, and thereby to raise the assessment from £170 to £3,170. Very great dismay has been caused by this statement, which, as Mr. R. Jewson, the chairman of the board, pointed out in a singularly able speech, has been increased by an unfounded charge made by a Guardian recently that the hospital "never has any nurses" when the infirmary had need of them, although the Guardians were prepared to pay for their services. That a request for nurses had had to be refused Mr. Jewson admitted, but for the sufficiently good reason, from which the Bishop, a member of the staff, and a local practitioner had also suffered, that there was not one nurse available. If the new assessment be enforced, the hospital's contribution to the rates will be raised from £77 to £1,408. Answers to inquiries have been received from two hundred hospitals, and enforce the "almost universal practice" that preferential treatment is given to hospitals in the matter of rating. That the hospital renders very important services, which would fall on the Guardians if its doors were closed, cannot be denied, nor can the fact that indoor recommendations are given to the Guardians in return for their subscription of fifty guineas, recommendations the financial worth of which can be precisely calculated from the cost of each person per diem who has been admitted by their means. Why, then, this sudden assault on the hospital's funds? It is argued ingeniously that the change in assessment is a purely administrative measure, and that it will be compensated by a corresponding increase in the Guardians' grant; that, in short, indirect rate-aid will be replaced by direct subsidy. If this be the Guardians' intention, and it is a plausible one, why was it not made clear at the time? For the resolution apparently was as clear about the rise in the assessment as it was silent about a rise in the subsidy. There is yet time, however, for the Guardians to escape from the unique and disastrous position in which they have placed themselves, by making it quite clear that their grant will be correspondingly increased. If this be not done, only one motive beyond active hostility to the hospital can be suggested for their action, that, namely, it was an attempt to press the hospital to induce the County Council to make a grant in return for the patients which the hospital receives from all its borders. We are loath to think the Guardians so unstatesmanlike as to have recourse to such a round-about policy. We are still more loath to see them stigmatised as the hospital's foes. It is for them to explain their action.

### HOW TO IMPROVE MEDICAL EDUCATION.

The opening address given by Dr. Seymour Taylor at West London Hospital on Monday last raised again the old problem of the want of organisation in the medical teaching of the Metropolis. "If the hospitals of London were properly organised," the speaker said, "there would be no necessity for a doctor to go to Paris or Berlin. We have as much clinical material here. But many scattered hospitals, being under different management and control, were effective obstacles to an advance of medical teaching." If London was to acquire its proper status as a centre of medical education, the machinery would have to be brought up to date. Ten or twelve medical schools were not required in London. Let these, or some of them, amalgamate, and let the beginning already made continue. As far as teaching was concerned, he wanted to know where was the wisdom of having three separate hospitals on the south side of the Thames. And the speaker concluded with the comprehensive suggestion that a building should be erected somewhere near the "Elephant and Castle," at a point, that is to say, almost equidistant from Guy's, St. Thomas's, and the new site of King's College Hospital.

### DOCTORS' FEES AT INQUESTS: A SUGGESTION.

The question of doctors' fees for giving evidence at inquests was raised once more at the Southwark Coroner's Court last week, when Dr. Schlesinger asked the City coroner if the jury would make some recommendation. The City coroner replied that he thought the doctors ought to be remunerated, but that he was bound by Statute law and by the regulations of the London County Council. A private doctor was paid one guinea for attendance and one guinea for a post-mortem, but the coroner was not allowed to give a hospital doctor any fee. In 1909, the City coroner recalled, a Home Office Departmental Committee had recommended in their report a change in the law. He advised the doctors to ask the members of Parliament for Southwark and any others known to them to press the Home Secretary to bring legislation in accordance with the suggestions of the committee. At present all that could be done was for the jury to make a recommendation. This, however, was not made, the foreman stating that he was much in sympathy with the poor ratepayers. That point of view, however, does not affect either the anomalies of the present law or the fact that expert work should not be expected of the hospital doctor without a fee because he happens to be a member of a hospital staff. The principle and the work are the same in both cases, and that they have been recognised in the one may be taken as a sign of the twinges of conscience which the Legislature has experienced from time to time on this question.

### ST. MARY'S NEW CASUALTY DEPARTMENT.

The opening of the new casualty department of St. Mary's Hospital sees the latest addition to that structural development which has been going on in this branch of hospital work in this part of London. The Children's Hospital at Paddington Green has now a complete out-patient unit, and St. Mary's at length has followed suit by the reorganisation of the eastern part of the building. The outline of the scheme has been given in these columns already, and we need only remind our readers how absolutely necessary it was to replace the old-fashioned and dreadfully cramped quarters in which casualty cases had to be treated with others of more modern capacity, design, and convenience. Indeed, the reconstruction had been postponed to the latest possible minute, and the visitor will now rejoice in the rearrangement by which the architect has converted the old enormous board room and the adjoining apartments into a waiting hall, a surgery, and anæsthetic, medical, infectious, and isolation rooms. Some £1,500 of the £8,000 required has yet to be subscribed, and, as we pointed out when describing the conditions on which the appeal was based in May 1911, this appeal is an appeal for the patients if ever there was one. It must be with a sigh of relief as well as pleasure that the hospital authorities contemplate to-day the undisastrous end of those risks which were inseparable from the cramped conditions of the old casualty department.

### THE INSURANCE ACT'S EFFECT ON HOSPITAL SATURDAY.

Though it is too early yet to give the precise sum which the Hospital Saturday collection has realised this year, Mr. A. W. Davis, the secretary of the Fund, has already expressed his fears that last year's total of £45,468 will not be reached.

Up to Midsummer, he remarks, the income was nearly £600 greater than that for the corresponding period of last year, but since July an ominous decrease has occurred which has annulled this increase, and, indeed, reduced the income in addition by £200. The penny weekly subscriptions on the cards of many factories and workshops began to fall off directly the contribution cards of the Insurance Act came into operation. When the benefits under the Act become available Mr. Davis hopes that the withdrawals will be made good.

### SARAH BERNHARDT AT CHARING CROSS HOSPITAL.

On Tuesday afternoon Madame Sarah Bernhardt paid a memorable visit to Charing Cross Hospital. On her arrival, soon after a quarter-past two, the great actress was received by the Chairman, Mr. Robert Duff, Mr. Walter Alvey, and several members of the council and the medical and surgical staff. After graciously posing to be photographed as she received a bouquet at the hands of a young patient, Dorothy Hunt, who has recovered from an operation for hernia, Madame Bernhardt went up by the lift to the top floor, where she spent some time in the Levy Ward, chatting in particular to two patients. From this ward she passed to the *x*-ray room, where several photographs taken the previous day were examined, and then Madame Bernhardt was persuaded to allow an *x*-ray photograph of her hand to be taken as a memento of her visit. This comes with delightful appositeness after the remarks made in The Hospital three weeks ago on Madame Bernhardt's interest in hospitals in general. Her visit to Charing Cross Hospital, which was carried out with the leisure that comes of a keen interest in all the objects seen, will long be remembered by all present, from the cheers of the students in the hall as Madame Bernhardt entered in a striking costume of yellow and sables, to the last smile she gave as she left the building. May Madame Bernhardt's interest be reflected in the public and in the progress of the institution which is in great financial need!

### THE PROPER HOUSING OF THE DOMESTIC STAFF.

Important alterations will be be made to the Cameron Hospital, West Hartlepool, as a result of Alderman Wilson's unexpected and generous gift of £1,000. How badly improvements are needed is seen from the fact that the maids at the present time are accommodated in rooms originally intended, as Mr. M. Harwood has pointed out, for box rooms. In addition it is felt that the nurses' quarters are inadequate, and that a new ward is required. A new wing is, therefore, a summary of the general needs of the hospital, to which Alderman Wilson's gift will give an inspiriting start, while from time to time the provision of proper fire escapes and of a sterilising room have been under discussion. The maids' quarters, however, appear to need the first attention. Many institutions, we fear, still exist in which inadequate attention is paid to the comforts of the domestic staff, though there is perhaps no better indication of a hospital's general efficiency than their contentment and well-being. It was some time before the importance of giving to every nurse a separate room of her own was generally recognised, and of accommodating the nursing staff away from their work in a separate nurses' home. The day is coming when the needs of the domestic staff will be recognised by analogous treatment. Indeed, one or two institutions could be mentioned at which what is virtually a domestic home is in existence. That, no doubt, is the line of progress, and Alderman Wilson will deserve the thanks of every hospital worker if his gift is made the means of placing the domestic staff of the Cameron Hospital under more up-to-date conditions than they are experiencing at present.

### DISUSED WORKSHOPS AS MENTAL WARDS.

As a temporary measure pending the erection of its eleventh mental hospital the London County Council intends to adapt two disused workshops at Colney Hatch Mental Hospital for use as dormitories. At present there is considerable pressure on the accommodation of the London County mental hospitals, and the alteration at Colney Hatch will provide additional sleeping accommodation for thirty-six male patients, day accommodation for whom can be found in existing wards. The cost to which the county would be put if this accommodation were not utilised and contract accommodation for thirty-six patients had to be found at out-county mental hospitals for the next four or five years would not be less than £360 a year. In view of the urgent need for the accommodation the approval of the Secretary of State to the plan of the structural alterations has already been obtained.

### STAFF DEVELOPMENTS AT ST. BARTHOLOMEW'S.

With the expansion of electrical and *x*-ray work which has been proceeding so rapidly in the last four or five years comes also the need for staff changes in the case of large hospitals where the department has outgrown the capacity of a single officer. Thus at St. Bartholomew's Dr. Hugh Walsham, so well known for his excellent work in this region of specialism, now becomes medical officer in charge of the *x*-ray department, and Dr. E. P. Cumberbatch assumes the separate title of medical officer in charge of the electrical department. Another new departure has been taken at the same institution by the appointment of Mr. R. C. Elmslie to be surgeon in charge of the orthopædic department. At too many of our leading hospitals orthopædics is so little regarded that one of the (general) assistant surgeons assumes a fictitious supervision of orthopædic cases, which are in reality dealt with just as they happen to come among the ordinary surgical out-patients by the officer of the day. There is no doubt that the change thus inaugurated at St. Bartholomew's is a step forward, and that similar changes are bound eventually to be made at all hospitals which desire to be abreast of the surgical times. For their own sakes, and for the reputation of the voluntary system in general, we trust that other hospitals will follow the example set at St. Bartholomew's.

### HOSPITALS AND SUNDAY ENTERTAINMENTS.

More than one hospital of late appears to have been put into difficulty by the promoters of Sunday concerts who have advertised them as on behalf of the institution without previously gaining permission of the hospital authorities. Now this sort of indiscretion, if it be no worse, deserves the severest censure. The directors of the Arbroath Infirmary being recently placed in this position retaliated in the only effective way possible to them, by declining to accept the proceeds of the offending entertainment. So long as the present confusion of opinion exists as to what amusements are allowable and what to be discouraged on Sunday, it is always very difficult for a charitable institution to take the responsibility of being publicly associated with any new departure of this kind. Local supporters must be unanimous on the fitness of such entertainments, whatever views may prevail in other localities. But the occurrence of advertisements associated with the names of institutions which have not given their consent to the announcement leads us to believe that here, too, is an evil, as in the case of the cinematograph interest. Exactly who is making money out of these Sunday concerts is a matter for investigation, but we suspect that the expenses incidental to them are ominously heavy.

### A PUBLIC MEDICAL SERVICE FOR SALISBURY.

One interesting effect of the Insurance Act has been the impetus which in some cases it has given to local effort, which is now expressing itself in various proposals for dealing with the problem created by the expected resignations of the doctors in January next. One of the latest and most interesting examples is the scheme which was adumbrated at a meeting of the governors and subscribers of the Salisbury and South Wilts Provident Dispensary last week. Alderman Hammick, the chairman, stated that the doctors had proposed to the dispensary's committee a scheme for a public medical service for treating persons not insured under the Insurance Act. The doctors, he explained, offered to rent the dispensary, and the rent might be devoted to the provision of treatment for the sick. Dr. Kempe, another speaker, explained further that the wage-limit for those wishing to participate had been fixed at two pounds a week. The subscription for uninsured persons would be twopence a week if the income exceeded one pound, and one penny halfpenny if it did not exceed one pound a week. For those who wished their wives and families to be included a tariff had been drawn up. The speaker concluded by saying that the conditions of service embodied all the claims of the British Medical Association. The committee decided to enter into negotiations with the doctors on the above lines. The scheme's progress will be watched with interest.

### MUSIC AND A BIRMINGHAM HOSPITAL.

The recent holding of the Triennial Musical Festival at Birmingham is of interest not merely because it was the forty-fourth occasion on which the General Hospital had been associated with it, but for the regrettable assertion that it may be the last of a series which has become famous both in musical and in hospital annals. The attendance has dwindled, and the question has arisen whether or not the Festival shall be repeated. The first was held in 1784, and originated even earlier in the musical entertainment which was established to aid the funds of the General Hospital which was then in process of foundation, though not actually established till 1799. Of the musical success of the Festivals and of their fine record it is unnecessary to speak here, but even this can be guessed from the fact that the General Hospital has benefited by them to an amount which runs well into six figures. It will certainly be unworthy of so enterprising a city as the Midland capital undoubtedly is if the Festival is allowed to lapse because fashions have changed, or, perhaps, because an influx of new blood and enthusiasm is needed. If there were enough enterprise sixty years ago to secure Mendelssohn to conduct in person, there should be enough to-day to secure Strauss or Debussy, or whomever the musical enthusiasts may most desire to hear. The Festival should not be allowed to pass away without an effort, and this, the finest example of the co-operation between music and charity, which has been illustrated often in our columns, demands the support of every Midland hospital worker that a great tradition may be handed on unimpaired.

### RELIGION AND SANITATION.

The religious customs of the non-Christian portion of the Empire are generally brought into public notice by the problems of hygiene, health, or sanitation which they raise. The latest example of this is the comprehensive survey which the Government of India is instituting into the best methods of improving the sanitary arrangements at the various centres where Mohammedan and Hindu pilgrims meet for starting on their journeys to Mecca or for other religious purposes. The number of such pilgrims has increased so enormously with modern travelling facilities that there is always a serious risk of the importation as well as of the outbreak of disease at these centres. The inquiry is expected to co-ordinate the arrangements which have already been enforced at some of the more important centres. Each province will have the benefit of the Imperial Sanitary Commissioner's presence, who will draw up a report on the result of the local inquiries. On each local committee will be a representative of each railway which is engaged in the pilgrim traffic. These facts have peculiar interest as showing how Western science complicates at first Eastern religious customs by making their observance possible to even larger numbers of the devout, and then by regulations necessary for the public health may end in modifying them to a degree not foreseen or contemplated.

### A NEW POST FOR A POOR-LAW PHARMACIST.

Many readers will remember that in May last we referred to the innovation of electrical apparatus at St. James' Infirmary, Wandsworth Common, and to Dr. Miller's—the medical superintendent's—expression of satisfaction with the progress which Mr.

W. E. Kinsman, M.P.S., had made in that department. At the last meeting of the Wandsworth Board of Guardians the recommendation of the finance committee, that the good work performed by Mr. Kinsman was worthy of recognition, culminated in the Board agreeing to create a post of "*x*-ray worker," at a salary of £25 per annum, subject to the approval of the Local Government Board. The value of the scientific training of the pharmacist is thus brought into prominence, and we congratulate the medical superintendent and the Guardians for their recognition of merit. The adaptability of the pharmacist has been well proved by Mr. Kinsman.

### GRIMSBY HOSPITAL AND THE EDUCATION AUTHORITY.

The management committee of the Grimsby and District Hospital has adopted a resolution asking for a grant from the education authority in respect of the operations performed by the hospital on the school children. The chief speaker at the meeting which came to this decision was Mr. J. Waddingham, the secretary of the Working-men's Hospital Committee, who for two years has been endeavouring to secure a grant of this character. He said that the education authority either must provide a grant or open a school clinic of its own. During the year ending July 31, 1911, contributions to hospitals were made by thirty-four authorities. As the Act was permissive only, the education authority must not forget that they had a moral obligation in the matter. Councillor Whiteley Wilkin, on the other hand, contended that, though the number of adenoid and tonsil cases had increased by some 600 since medical inspection took place, the average increase of cases treated at the hospital was only 94. This probably meant that the hospital was treating only the children of those who could not afford to have them treated by their own medical men. The matter requires careful investigation, but it will be very surprising if Grimsby proves the one centre where no extra work has been cast on the hospitals through the medical inspection of school children.

### SANATORIUM BENEFIT IN DERBYSHIRE.

Among the latest local schemes for the administration of sanatorium benefit under the Insurance Act is that which the Derbyshire County Council adopted last week. The County Insurance Committee expressed its willingness to enter into a contract for the treatment of insured persons which would not incur any charge on the county rate, while the County Council was to be responsible for persons who were not insured and who are to be charged such fees as they can afford. Thirty-five beds are to be reserved for Derbyshire in a sanatorium which will be shared jointly with county boroughs, three branch sanatoria in the neighbourhoods of Chesterfield, Chinley, and Ilkeston, and fifty beds at certain isolation hospitals which will be used chiefly for advanced cases. There will also be local and branch dispensaries. It is understood that the County Council's share of the Government grant towards structural expenditure will be £18,000, while £11,000 a year for maintenance will be provided by the County Insurance Committee. It is generally believed that such sums will be ample to meet the necessary expenditure.

### NEW NURSING HOME AT CHISWICK.

Last week Sir Anthony Bowlby, C.M.G., opened the St. Mary's Nursing Home, Chiswick, an expansion of the work of the St. Joseph's Hospital for Incurables in the same parish. The hospital itself is attached to St. Mary's Convent in Burlington Lane, Chiswick, and is now under the care of a sisterhood connected with East Grinstead. It was originally the property of the community of St. Mary and St. John, and was established in 1869 in Kensington Square. Rather more than two years ago the hospital was transferred to the present management; it receives especially female patients suffering from the various forms of paralysis and paralytic affections, rheumatoid arthritis, and similar diseases; also cripples and old people suffering from senile decay. The new nursing home has quite a different scope, although it is installed in the same building as the hospital, of which, indeed, it occupies the upper part. The home is intended for paying patients, and the buildings have been entirely remodelled to provide the necessary accommodation. We note that there is still a debt of £6,000 to be paid off, the balance of the cost of additions and improvements.

### AN APPOINTMENT AND A VACANCY.

It is announced that Mr. Archibald Donald, M.D., M.R.C.S., has been appointed to the Professorship of Obstetrics and Gynæcology at Manchester University, vacant through the death of Professor Sir William Sinclair. Dr. Donald is an Edinburgh graduate, is gynæcological surgeon to the Manchester Royal Infirmary, and is the author of an "Introduction to Midwifery." The governors of the London Hospital are advertising for an obstetric physician in place of Dr. A. H. N. Lewers, who has resigned. Dr. Lewers has rendered long and loyal service to the London Hospital, but his resignation is somewhat in the nature of a surprise; his term of office would expire under the regulations in about five or six years, we believe. The assistant obstetric physician, whose promotion is of the nature of a foregone conclusion, is Dr. H. R. Andrews. In the event of his being elected in Dr. Lewers' place, the junior post becomes vacant. The obstetric tutor and registrar is Dr. R. D. Maxwell, who is the next senior London Hospital man in this specialty. In Dr. Lewers' case the electors passed over the local candidates, for he was educated at University College Hospital, but it is improbable that any imported candidate will be selected this time.

### A NEW USE FOR THE HOSPITAL REPORT.

A curious communication came before the monthly meeting of the Batley and District Hospital last week. A man from London wrote saying he was prepared to sell the "names and addresses of charitably disposed persons residing in London and the provinces." He added that "such lists would be found useful for appealing for funds,"

and was prepared to retail them at two shillings and sixpence per hundred, or, if over one thousand were ordered, for the reduced fee of two shillings for every additional hundred till a second thousand had been reached. The hospital's Secretary, Mr. A. W. Western, then explained that he had frequently received applications from private persons for copies of annual reports, and had been curious to know the reason. The above letter he thought provided an explanation, which led him to inquire whether he should send copies to such persons when requested in future. One gentleman present hastily replied, "Certainly not." We hope that was not the general feeling of the meeting. A hospital secretary's duty is to see that his institution's annual report has the widest circulation obtainable, and to act on the hypothesis that every stranger who applies for a copy has done so for the purpose of offering for sale the names of the subscribers to institutions in other parts of the country would be to run the very serious risk of offending an intending benefactor. It is impossible to exercise any control over the uses to which reports may be put by those who apply for them. We cannot prevent them from being thrown unread into the waste-paper basket; but that fear leads energetic secretaries not to restrict their circulation but to take more trouble and pains to make them attractive reading. The moral is the same in this case.

### HOUSE OFFICERS UNDER THE ACT.

The Medical Defence Union has succeeded in its attempt to get the Insurance Commissioners to investigate the question whether members of the resident medical staffs of hospitals are employees under Section 66 of the National Insurance Act. The Commissioners have accordingly decided to hold an open inquiry on October 22 next at 2.30 p.m. at the Civil Service Commission Offices in Burlington Gardens. It is important that all sides should be represented at this inquiry, and hospital workers as well as resident medical officers who have any views on the subject would do well to write to the Commissioners and ask to be heard on the point. There are many arguments against the view that medical house officers are employees in the sense of the Act, and the position taken up by the Medical Defence Union is a strong one on which it has decided to raise a test case, with a subsequent appeal to the High Court if the Commissioners do not admit its contention. The Union urges that there is no contract of service, and that it is unfair to compel house surgeons and house physicians to comply with the regulations of the Act when they stand no chance of benefiting by its provisions. Hospital authorities are concerned in the question and would be well advised to be represented at the inquiry either by their solicitors or by some members of their house committees.

### THE GROWING CLAIM ON WOMEN PRACTITIONERS.

An eloquent appeal on behalf of the proposed South London Hospital for Women, the details of which are discussed in our leading article, has been published by Lady Robert Cecil, in the course of which she reminds the general public of one or two facts which they do not apprehend. The first is how large the demand is among women to be treated medically by members of their own sex. It is common knowledge that women doctors are busy doctors and an increasingly numerous branch of the profession, but the complementary fact is not grasped that this increase is due not merely to the growing desire among women to enter the professions and to lead self-supporting lives, but at least equally to the growing demands for their services in medicine. Lady Robert is probably not far wrong when she affirms that among working-class women especially minor complaints are allowed to develop into major ones owing to their diffidence about consulting a medical man. Those still sceptical should test this argument by comparison with the demands on and the waiting lists of the New Hospital for Women.

### DISPENSING IN UNTRAINED HANDS.

The activities of the Public Pharmacists' and Dispensers' Association are being directed into just the channels which should attract the chemist engaged or interested in institution affairs. The inaugural address is to be given on October 30, by Sir Victor Horsley, who has chosen for his topic "Legislation of to-day and its effect upon the practice of pharmacy and medicine." This must form an excellent subject which such an authority ought to render extremely fascinating. The Association has addressed a communication to the Merthyr Guardians concerning their suggestion that a nurse should receive three months' tuition in dispensing in order to compound the medicines required in the workhouse. Such a preposterous idea must be killed by an appeal to public opinion should other means fail. We do not apologise for repeating our opinion that where compounding is a part of the duties, however small, of an officer, it must be performed by an expert. If there is less work than will occupy the full time of the pharmacist, then let duties of a less specialised nature be added. There is a great difference between having a qualified pharmacist performing secretarial duties and having a clerk or nurse dabbling with drugs.

### THIS WEEK'S DRUG MARKET.

There has been a noticeable improvement in business accompanied by several alterations in prices. The Opium market is still very unsettled, with the prospect of a further advance in price; morphine also tends dearer, and an announcement of an advance in the price of codeine should not come as a surprise. Menthol being scarce has risen in price considerably. A fair business has been done in camphor. Epsom salts have been slightly advanced in price. Ipecacuanha is dearer. Orris root has again been advanced in price.

### TO OUR READERS.

Contributions are specially invited from any of our readers to these columns. They should deal with topical subjects and news. They must be authenticated for the information of the Editor only. The minimum payment if published is 5s. There is no hard-and-fast rule as to space, but notes of about twenty lines in length are preferred.

